# Objective Treatment Targets and Their Correlation with Patient-Reported Outcomes in Inflammatory Bowel Disease: A Real-World Study

**DOI:** 10.3390/jcm14134733

**Published:** 2025-07-04

**Authors:** Panu Wetwittayakhlang, Siripoom Ngampech, Saichol Pattarakulniyom, Peter L. Lakatos

**Affiliations:** 1 Gastroenterology and Hepatology Unit, Division of Internal Medicine, Faculty of Medicine, Prince of Songkla University, Hat Yai 90110, Songkhla, Thailand; wet.panu@gmail.com (P.W.); siripoomtonza@gmail.com (S.N.); 2 Division of Internal Medicine, Faculty of Medicine, Prince of Songkla University, Hat Yai 90110, Songkhla, Thailand; bung.element@gmail.com; 3 Division of Gastroenterology and Hepatology, McGill University Health Centre, Montreal, QC H3G1A4, Canada; 4 Department of Internal Medicine and Oncology, Semmelweis University, H-1085 Budapest, Hungary

**Keywords:** Patient Reported Outcomes, Inflammatory Bowel Disease, Ulcerative Colitis, Crohn’s Disease, Treat to Target, Biomarker, Endoscopic, Biologic Therapy

## Abstract

**Background & Aims**: treat-to-target approach is essential for improving outcomes in inflammatory bowel disease (IBD). This study aimed to assess real-world achievement in objective monitoring (clinical, biomarker, and endoscopic assessments) and the correlation between patient-reported outcomes (PROs) and treatment targets. **Methods**: This retrospective study included consecutive IBD patients from January 2020 to December 2024. Disease activity was assessed using the Harvey-Bradshaw Index (HBI), partial Mayo score, PRO2, and PRO3, along with C-reactive protein (CRP) levels and endoscopic scores (SES-CD, MES). Clinical outcomes were evaluated at baseline, 1 year, and 2 years. **Results**: Among 112 IBD patients (55% with CD, median age at diagnosis: 45.2 years), clinical remission rates at baseline, 1 year, and 2 years were; CD: 75.8%, 70.0%, and 55.8%; UC: 84.0%, 79.5%, and 81.4%. CRP normalization rates at the same time points were; CD: 54.8%, 41.7%, and 63.8% UC: 78.0%, 70.5%, and 81.8%. Endoscopic remission rates were; CD: 58.1%, 50.0%, and 50.0%, UC: 71.4%, 64.5%, and 51.7% Flare-ups were more frequent in CD than in UC (32% vs. 20%), with an 8.1% rate of IBD-related surgery. In CD, PRO2 and PRO3 strongly correlated with clinical remission (AUC = 0.885 and 0.881), moderately with biomarkers (AUC = 0.737 and 0.755), and modestly with endoscopic remission (AUC = 0.695 and 0.685). In UC, PRO2 showed a strong correlation with clinical remission (AUC = 0.972) and moderate correlations with biomarkers (AUC = 0.653) and endoscopy (AUC = 0.783). **Conclusions**: Clinical remission was more frequent in UC than in CD. PROs showed a strong correlation with clinical remission but only moderate associations with biomarkers and endoscopic remission in both CD and UC.

## 1. Introduction

Inflammatory bowel disease (IBD), comprising Crohn’s disease (CD) and ulcerative colitis (UC), is a chronic immune-mediated condition of the intestines that can lead to irreversible intestinal damage and significantly impact patients’ quality of life [1]. While IBD has historically been most prevalent in Western countries, recent analyses reveal a growing incidence in newly industrialized nations, particularly in Asia [2]. The pathogenesis of IBD is multifactorial, involving a complex interplay between genetic predisposition, environmental factors, and an aberrant immune response, leading to chronic intestinal inflammation [3,4].

In the past decade, treatment goals for IBD have shifted from merely alleviating symptoms to achieving objective inflammatory control through treat-to-target strategies. This paradigm was first proposed in 2015 by the Selecting Therapeutic Targets in Inflammatory Bowel Disease (STRIDE) consensus [5,6]. The treat-to-target approach aims to achieve remission based on clinical symptoms, inflammatory biomarkers, and endoscopic mucosal healing, which have been associated with improved long-term outcomes and quality of life for IBD patients [5,6].

Endoscopic mucosal healing has been established as the ultimate long-term goal of IBD management. However, achieving this target requires intensive therapy, significant costs, and frequent endoscopic monitoring. In the short to intermediate term, symptomatic resolution remains a key treatment objective. To assess clinical response objectively, multiple symptom-based scoring systems have been developed for disease activity monitoring. The partial Mayo score (pMayo) is commonly used to assess UC activity, while the Harvey-Bradshaw Index (HBI) and Crohn’s Disease Activity Index (CDAI) are widely utilized for CD [7]. Despite their widespread use, these scoring systems have limitations, as they were originally developed for clinical trials and lack standardization for routine clinical practice. Some components, such as physician-reported global assessments, may not accurately reflect patients’ perceptions of their disease status, leading to interobserver variability.

In recent years, patient-reported outcomes (PROs) have emerged as essential tools for assessing disease activity and guiding treatment decisions [8]. PROs offer a broader understanding of a patient’s condition beyond traditional disease measures. Their integration into clinical practice promotes healthcare “coproduction,” fostering collaborative patient-provider relationships and enhancing shared decision-making, patient satisfaction, and clinical outcomes [9]. In addition, PROs help physicians identify patients likely to have active disease and prioritize them for endoscopic evaluation. The PRO2 score is a simplified tool for evaluating IBD symptoms: in UC, it consists of stool frequency (SF) and rectal bleeding (RB) scores, while in CD, it includes SF and abdominal pain scores [10]. According to the STRIDE-II consensus, treatment response is defined as a ≥50% reduction in PRO2 score, with clinical remission defined as RB and SF scores of 0 in UC and abdominal pain ≤1 and SF ≤3 in CD [6]. Several studies have showed a strong correlation between PROs and endoscopic remission in UC [11], while in CD, PROs correlate less reliably with the degree of mucosal inflammation [12]. More recently, a three-item PRO (PRO3) was introduced for CD, incorporating general well-being alongside SF and abdominal pain to better capture patient perception of disease activity and quality of life [10].

Although the STRIDE initiative strongly recommends the treat-to-target approach to improve IBD outcomes, real-world implementation remains challenging and varies globally. PRO remission has been found to be the most achievable target, whereas endoscopic and histological remission are more difficult to attain in routine clinical practice [13]. The PODCAST-IBD study, a real-world cross-sectional analysis in the UK and Spain, found that approximately 50% of CD patients and 40% of UC patients had suboptimal disease control [14,15]. Several factors may influence the achievement of treatment targets, including the affordability of biologic therapies, adherence to objective treatment strategies by both patients and physicians, and variations in patient perceptions of disease activity, which may impact PRO measurement.

Therefore, this study aims to evaluate the real-world achievement of treat-to-target objectives as recommended by STRIDE and to assess the correlation between PRO scores (PRO2 and PRO3), biomarkers, and endoscopic remission in patients with IBD.

## 2. Materials and Methods

### 2.1. Study Design and Population

This retrospective cohort study was conducted at Songklanagarind Hospital, Hat Yai, Songkhla, Thailand. We included all patients aged over 15 years who were diagnosed with IBD, including CD or UC, and who were either newly diagnosed or referred to the IBD clinic between 1 January 2020, and 31 December 2024. The diagnosis of IBD was based on a comprehensive assessment of clinical presentation, endoscopic findings, radiological imaging, and histopathological evidence, in accordance with the Lennard-Jones and Copenhagen diagnostic criteria.

This study utilized data from our institutional IBD clinic cohort registry, where patient-reported outcomes (PROs) have been systematically collected for all patients since 2020 in accordance with STRIDE recommendations. We included all patients who met the inclusion criteria and had no exclusions—this included both newly diagnosed patients and those previously diagnosed with IBD who were referred for continued care and had follow-up visits at the IBD clinic between 2020 and 2021. For each patient, the first visit with documented PRO assessment was defined as the baseline. PROs were subsequently assessed at each follow-up visit.

All patients with a confirmed diagnosis of CD or UC, a baseline PROs assessment, and at least two calendar years of follow-up were screened and enrolled. Clinical outcomes including clinical remission status, biomarker levels, endoscopic activity, need for intestinal surgery, hospitalization, and disease flare events were comprehensively reviewed. Patients were excluded if they had indeterminate colitis, did not meet diagnostic criteria for CD or UC, had less than two years of follow-up, or lacked baseline PRO data. This study specifically analyzed the associations between PRO scores (PRO2 and PRO3) and objective clinical outcomes—including partial Mayo score, Harvey-Bradshaw Index (HBI), biomarkers, and endoscopic activity, at 1 and 2 years following baseline. All eligible cases were independently screened by two investigators using predefined inclusion and exclusion criteria.

### 2.2. Data Collection

Baseline patient data, including patients’ demographics, date of IBD diagnosis, disease duration, disease phenotypes, history of intestinal surgery, and medical therapies were collected from the electronic medical record. Disease extent, severity, and behavior were classified according to the Montreal criteria [16]. In addition, biomarkers including C-reactive protein (CRP) and erythrocyte sedimentation rate (ESR), along with endoscopy were assessed. Disease activity was assessed using clinical scores (Harvey Bradshaw Index [HBI] for CD and partial Mayo score [pMayo] for UC), as well as endoscopic activity (Simple Endoscopic Score for CD [SES-CD], endoscopic Mayo score [eMayo], and Endoscopic Index of Severity [UCEIS] for UC).

PROs score was reported directly by patients using harmonized questionnaire record form. In UC, PRO2 were calculated from rectal bleeding and stool frequency. In CD, PRO2 was calculated from stool frequency and abdominal pain, and PRO3 was calculated from stool frequency and abdominal pain, and general well-being (Supplemental Table S1). In patient with multiple visit, PROs score was calculated at closest to endoscopic evaluation. The PROs, clinical and biochemical and endoscopic assessments were captured at baseline (at the first visit at IBD clinic after initiation of the study), 1-year, and 2-year after the initial evaluation. All information was recorded in electronic case record forms.

### 2.3. Definition of Study Outcomes

The co-primary outcomes were the achievement rates of treatment targets including clinical symptoms, biological markers, and endoscopic assessment. The secondary outcomes were the correlation of PROs scores and treatment targets including biomarker and endoscopic remission.

Clinical remission was defined as an HBI < 5 in CD and a partial Mayo score ≤ 2 in UC patients without episode of disease flare within 3 months. Biomarker remission was defined as CRP in below the upper normal limit (<5 mg/L). The endoscopic remission was defined as an SES-CD < 3 in CD and Mayo endoscopic score (MES) ≤ 1 in UC. Patients who did not have a clinical evaluation at each follow-up visit were considered not in clinical remission. Flare of disease was defined as a pMayo > 3 and/or RB subscore > 1 for UC or HBI ≥ 5 for CD in the patients previously in clinical remission.

### 2.4. Statistical Analysis

Descriptive statistics were used to summarize the study population. Categorical variables (e.g., gender, disease phenotype) were presented as frequencies and percentages. Continuous variables (e.g., age, biomarker levels) were reported as means with standard deviations (SD) for normally distributed data, or as medians with interquartile ranges (IQR) for non-normally distributed data. Normality of distribution was assessed using the Shapiro–Wilk test. Comparisons between CD and UC groups were performed using the Chi-square test or Fisher’s exact test for categorical variables, and independent *t*-tests or Mann–Whitney U tests for continuous variables, as appropriate.

The rates of achieving treatment targets—including clinical, biomarker, and endoscopic remission—were assessed at baseline, one year, and two years, and expressed as percentages based on the number of evaluable patients at each time point. Remission rates were calculated only among patients with available data at each respective time point. Patients with missing outcome data were excluded from the specific analysis, and no imputation was performed. A sensitivity analysis was conducted using complete-case data to assess the robustness of findings. Receiver operating characteristic (ROC) curve analysis was performed to evaluate the discriminatory performance of PRO2 and PRO3 scores in predicting clinical, biomarker, and endoscopic remission. ROC curves were generated using pooled data from all available time points per patient. The area under the curve (AUC) was reported, and the optimal cutoff values were determined using the Youden index. All statistical analyses were performed using R software, version 4.5.0.

## 3. Results

### 3.1. Baseline Characteristics of the Study Population

A total of 112 IBD patients were included, with 62 (55.4%) diagnosed with CD and 50 (44.6%) with UC. CD patients were younger at diagnosis (mean 43.6 vs. 47 years) and had a longer diagnostic delay (median 39.5 vs. 13 weeks). Baseline characteristics are summarized in Table 1.

In CD, the most common phenotype was ileocecal involvement, and 25.8% had stricturing or penetrating disease. Perianal disease was noted in 9.7%. In UC, extensive colitis (E3) was most frequent (68%), with over half presenting moderate to severe disease. Extra-intestinal manifestations were observed in 13.4% of the total cohort. Biomarker (CRP, ESR) were generally higher in CD. Median HBI and SES-CD scores at CD diagnosis were 5 and 6, respectively, while UC patients had a median partial Mayo score of 5.5 and MES of 2. Nearly all UC patients received 5-ASA (98% vs. 56.5% in CD), while biologics were more common in CD (27.4% vs. 14%). Steroid exposure was high in both groups, with steroid dependence more frequent in UC (50% vs. 37.1%). About 70% of patients in both groups were treated with thiopurines.

### 3.2. Real World Achieving Treatment Targets in IBD

**Figure 1:**
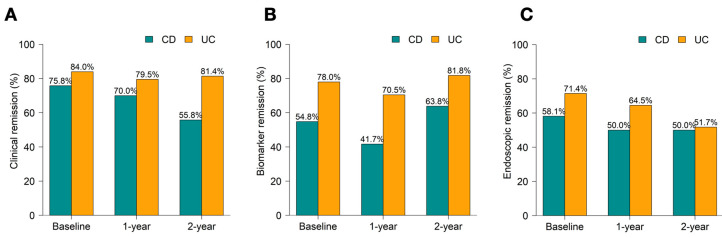
Rate of achieving in treatment targets for Inflammatory Bowel Disease (IBD) (**A**). clinical remission (**B**). biomarker remission (**C**). Endoscopic remission.

All patients were evaluated for PROs and biomarkers, with 69.6% undergoing baseline endoscopy. Follow-up adherence declined slightly: PROs were available in 92.6% at 1 year and 84.8% at 2 years; biomarker assessments in 92.6% and 81.8%, respectively; and endoscopic assessments in 56.3% and 50.9%. At baseline, clinical remission was achieved in 75.8% of CD and 84% of UC patients. Biochemical remission rates were lower—54.8% in CD and 78% in UC—while endoscopic remission was observed in 58.1% of CD and 71.4% of UC. The rate of achievement of treatment targets including, clinical remission, biochemical remission, and endoscopic remission, as shown in Figure 1 and Table 2.

Over time, CD patients showed a decline in clinical remission from 70.0% at 1 year to 55.8% at 2 years, while UC patients maintained high rates (79.5% and 81.4%). Biochemical remission remained consistently higher in UC (70.5–81.8%) than in CD (41.7–63.8%). Endoscopic remission declined in UC (71.4% to 51.7%) but remained relatively stable in CD (50% at both timepoints). Trend changes in clinical score, biochemical value and endoscopic score is detailed in Supplement Table S2.

### 3.3. Outcomes in Clinical Flare and Surgery

During the two-year follow-up period, the cumulative rate of disease flare-ups was 26.8% (30 out of 112 patients), with a slightly higher incidence in the CD cohort (32.3%) compared to the UC cohort (20.0%). Additionally, five patients with CD (8.1%) required surgery during the follow-up period.

### 3.4. The Correlation Between PROs and Clinical, Biomarker, and Endoscopic Remission

**Figure 2:**
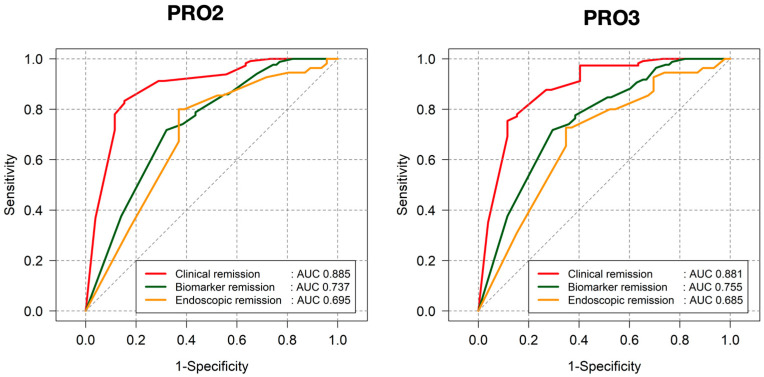
Correlation between patient-reported outcomes (PRO2 and PRO3) score and treatment targets in CD patients.

In the overall cohort, In CD, the PRO2 score showed a strong correlation with clinical remission, as assessed by the HBI score (AUC = 0.885), but demonstrated a moderate correlation with biomarkers (AUC = 0.642) and endoscopic remission (AUC = 0.596). Similarly, the PRO3 score exhibited a strong correlation with clinical remission (AUC = 0.881) and a moderate correlation with biomarkers (AUC = 0.634) and endoscopic remission (AUC = 0.580) (Figure 2).

Subgroup analysis based on clinical activity status in CD patients. In CD patients with clinical remission, both PRO2 and PRO3 demonstrated good discrimination for biomarker remission (AUC 0.769 and 0.760, respectively), while their performance for predicting endoscopic remission was limited (AUC 0.510 and 0.500, respectively). Among patients with active clinical disease, PRO2 and PRO3 maintained moderate discriminatory ability for both biomarker remission (AUC 0.686 and 0.720, respectively) and endoscopic remission (AUC 0.601 for both scores) (Supplement Figure S1).

In CD patients, the discriminative performance of PRO2 and PRO3 scores showed time-dependent variation, particularly for clinical and endoscopic remission. While both PRO2 and PRO3 demonstrated high AUCs for clinical remission at all time points (AUC > 0.85), a gradual decline was observed from baseline to year 1, with partial recovery by year 2. Notably, the AUCs for endoscopic remission fluctuated across time points (e.g., PRO2 AUC: 0.674 at baseline, 0.621 at 1 year, 0.830 at 2 years), suggesting that PRO performance may vary depending on disease phase and timing of assessment (Supplement Table S3).

**Figure 3:**
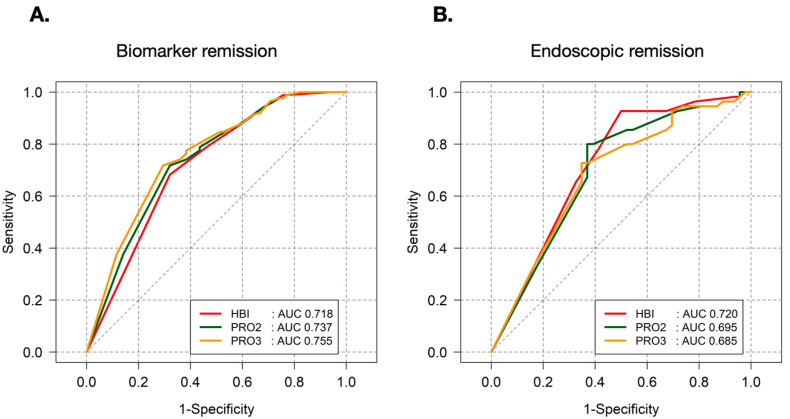
Comparison of HBI, PRO2 and PRO3 score in prediction of (**A**) biomarker remission and (**B**) endoscopic remission.

The optimal cutoff score for predicting endoscopic remission was found to be <6 for both PRO2 and PRO3. For PRO2, this cutoff demonstrated a sensitivity of 63.0% and a specificity of 80.0%, with a positive predictive value (PPV) of 72.5% and a negative predictive value (NPV) of 72.1%. For PRO3, the same cutoff score yielded a sensitivity of 65.2% and a specificity of 72.7%, with a PPV of 66.7% and an NPV of 71.4%. When compare to HBI, the PRO2 and PRO3 scores showed similar predictive power for biomarker remission and endoscopic remission (Figure 3).

**Figure 4:**
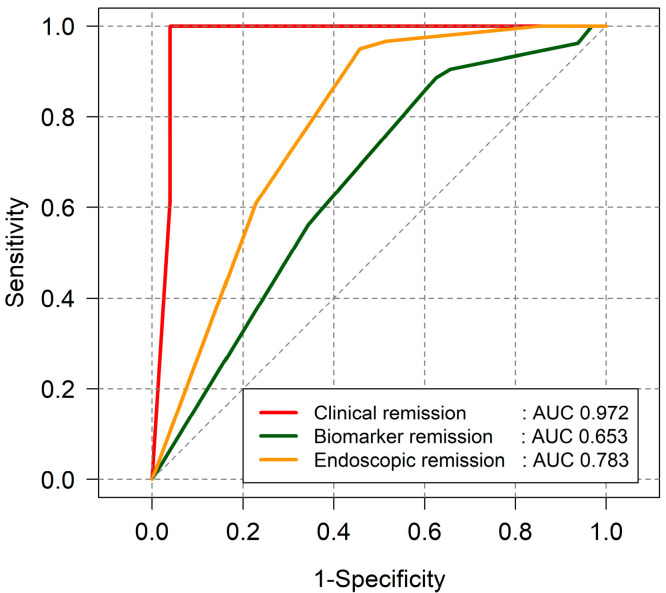
Correlation between PRO2 score and treatment targets in UC patients.

In UC patients, the PRO2 score showed a strong correlation with clinical remission, as assessed by the partial Mayo score (AUC = 0.972). However, its correlation with biomarkers (AUC = 0.653) and endoscopic remission (AUC = 0.783) was moderate (Figure 4).

In UC patients with clinical remission, PRO2 demonstrated a strong association with endoscopic remission (AUC 0.702); however, its ability to predict biomarker remission was limited (AUC 0.513). In patients with active clinical disease, the discriminatory ability of PRO2 was modest for both biomarker remission (AUC 0.540) and endoscopic remission (AUC 0.571) (Supplement Figure S2).

In UC patients, PRO2 and PRO3 showed strong and consistent predictive performance for clinical remission, with AUCs remaining high across time points (0.867 at baseline, 0.862 at 1 year, 0.818 at 2 years). Discrimination for biomarker remission was acceptable (AUCs: 0.709–0.768), while endoscopic remission showed moderate accuracy (AUCs: 0.686–0.796) (Supplement Table S4).

**Figure 5:**
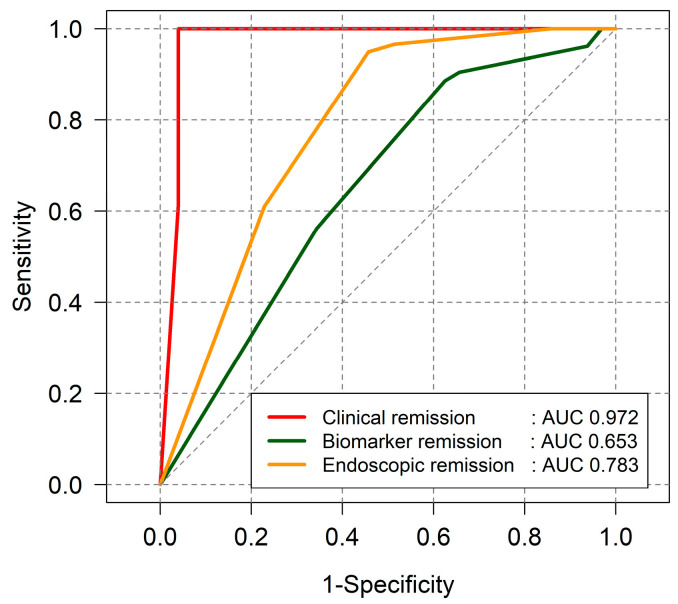
Comparison of partial Mayo and PRO2 in prediction of A. biomarker remission and B. endoscopic remission.

A PRO2 score cut off <1 score predicted endoscopic remission in UC patients with a sensitivity of 54.3% and specificity of 94.9%, yielding a positive predictive value (PPV) of 86.4% and a negative predictive value (NPV) of 77.8%. Additionally, both the partial Mayo score and PRO2 score exhibited comparable predictive power for biomarker and endoscopic remission (Figure 5).

## 4. Discussion

In this present study, we evaluated the achievement of objective treatment targets based on STRIDE recommendations by implementing a treat-to-target strategy in real-world IBD practice. Clinical remission was more frequently achieved in UC patients (80–84%) compared to CD patients (56–76%). Biomarker remission rates were also higher in UC (75–82%) than in CD (55–64%). Endoscopic remission was also more frequently achieved in UC than in CD. Additionally, our findings indicated that CD patients had a higher risk of disease flare-ups and need for surgery compared to UC patients.

Differences in treatment target achievement may reflect the distinct disease courses of CD and UC. In the population-based IBD South Limburg Cohort, only 50% of CD patients achieved remission over 10 years, with 6% exhibiting chronic continuous activity and 30% showing moderate-to-severe chronic intermittent disease [17]. In contrast, the IBSEN study found that 69% of UC patients had mild disease or were in remission 20 years post-diagnosis [18]. CD is also associated with a higher risk of complications, with one-third developing strictures, abscesses, or fistulas within 10 years and nearly half requiring intestinal surgery within 20 years [19,20,21,22]. Our study demonstrated a progressive decline in clinical remission rates among patients with CD over the two-year follow-up period, accompanied by an increased incidence of loss of therapeutic response. This may reflect a higher flare rate in CD, potentially linked to steroid tapering in corticosteroid-dependent patients. In line with the results from the IBD-PODCAST, a multicenter observational study conducted across 10 countries, reported the implementation of a treat-to-target strategy based on STRIDE criteria in real-world clinical practice that suboptimal disease control was identified in approximately 50% of patients with CD and 40% of those with UC [23,24].

Achieving endoscopic mucosal healing as a long-term treatment target remains one of the most challenging goals in IBD. In our study, a higher proportion of UC patients achieved endoscopic remission compared to CD patients. Notably, in UC, the rate of endoscopic remission declined over time, from 71.4% at baseline to 64.5% at 1 year and 51.7% at 2 years, whereas the rate remained stable in CD. In the SONIC study, endoscopic remission rates in CD patients treated with infliximab monotherapy and infliximab in combination with azathioprine were approximately 30% and 44%, respectively, at week 26 [25]. In contrast, endoscopic healing rates have been reported to be higher in UC than in CD. The ACT I and ACT II studies demonstrated mucosal healing rates of approximately 60% in UC patients treated with infliximab at week 8 [26]. Similarly, the GEMINI I study reported that 54% of UC patients treated with vedolizumab achieved endoscopic remission [27]. It is important to acknowledge that the patient characteristics in our cohort differed from those typically enrolled in clinical trials. Notably, the majority of patients were in clinical remission at baseline and has less severe disease, with only 20% required biologic therapy.

In addition, therapeutic strategies differed notably between CD and UC patients in our cohort, which may have contributed to the observed differences in treatment target achievement and PRO performance. Specifically, 98% of UC patients were treated with 5-ASA, compared to only 56.5% of CD patients. This reflects current guideline recommendations, as 5-ASA is the mainstay of treatment for mild to moderate UC but has limited efficacy in CD, especially for small bowel involvement. Conversely, the use of biologic therapies was more common in CD (27.4%) than in UC (14%), consistent with the generally more aggressive disease course in CD. However, the relatively modest clinical and endoscopic remission rates in CD may reflect delayed initiation of biologics, more complex disease behavior (e.g., stricturing or penetrating phenotypes), and variable therapeutic response. Furthermore, corticosteroid use was prevalent in both groups (87.1%) in CD and 90% in UC), but steroid dependence was more frequent among UC patients (50% vs. 37.1%), which may indicate differences in treatment responsiveness and tapering strategies. These medication-related differences likely influenced both short-term outcomes and the performance of symptom-based PRO tools, and should be considered when interpreting comparative results across IBD subtypes.

Our findings support the clinical utility of PROs for routine disease activity monitoring, as endorsed by STRIDE guidance. PRO2 and PRO3 scores were strongly associated with clinical remission and demonstrated moderate correlations with both biomarker levels and endoscopic remission in CD. Furthermore, PRO2 and PRO3 exhibited predictive performance comparable to the HBI in forecasting biomarker and endoscopic remission. These results are consistent with a retrospective analysis by Khanna et al., which validated the use of the PRO3 score in assessing CD activity, showing a moderate correlation with both the CDAI and the HBI [10]. While the correlation with endoscopic remission is more modest, the moderate performance in patients with active clinical disease suggests that PROs can serve as practical, non-invasive tools for monitoring disease trajectory and therapeutic response. In clinical settings where routine endoscopy or biomarker testing may not be feasible, PROs offer an efficient alternative for evaluating patient status and guiding treatment decisions. These findings support the integration of PROs into routine IBD care, especially for patient-centered monitoring and early detection of changes in disease activity.

In patients with UC, the PRO2 score showed a strong correlation with clinical remission and a moderate correlation with endoscopic remission, while its association with biomarkers was modest. Both PRO2 and partial Mayo scores were strongly associated with endoscopic remission. In UC, PRO2 correlated more closely with endoscopic than biomarker remission, particularly among patients already in clinical remission. Its performance was less robust in active disease, suggesting that symptom-based measures may be more reflective of mucosal healing in stable patients. Additionally, time-stratified sensitivity analyses demonstrated that the predictive performance of PRO2 and PRO3 for clinical remission remained consistently strong across all time points, particularly in UC. This stability over time reinforces the reliability of PROs in longitudinal disease monitoring.

A previous meta-analysis reported that a rectal bleeding subscore of 0 in the PRO2 score achieved high sensitivity (81%) for predicting endoscopic remission, while the combination of rectal bleeding and stool frequency subscores of 0 yielded a high specificity (96%) [11]. Furthermore, the resolution of diarrhea and the absence of rectal bleeding have been identified as independent predictors of relapse-free and colectomy-free survival, as well as favorable long-term outcomes in UC patients [28]. Jairath et al. evaluated the performance of RB, SF, and PRO2 in predicting endoscopic remission using pooled data from mesalamine induction trials. The area under the curve (AUC) values for RB alone, SF alone, and PRO2 were 0.78, 0.85, and 0.90, respectively, for predicting endoscopic remission defined as a Mayo endoscopic subscore (MES) ≤ 1 [29]. Additionally, a study by Dragasevic et al., including 159 IBD patients (63 with CD and 96 with UC), reported a positive correlation between PRO2 scores and both endoscopic and histologic activity in UC. In contrast, an inverse correlation was observed in CD, potentially due to the predominant small bowel involvement, which may result in the absence of rectal bleeding and preservation of normal stool frequency [30]. In our cohort, however, approximately 50% of CD patients had ileocecal involvement, and PRO scores still correlated reasonably well with both biomarkers and endoscopic activity.

Our finding supports the utility of PROs in disease activity monitoring regardless of disease location. However, PRO scores may be less accurate in IBD patients with coexisting irritable bowel syndrome (IBS), as increased PRO scores have been observed in a significant number of patients with increased stool frequency despite endoscopic remission. A meta-analysis showed that UC patients with persistent symptoms but minimal or no bowel inflammation may have a high prevalence of IBS, which is associated with worse PROs scores [11]. Similarly, a study on CD patients found that those with IBS had significantly higher pain sub-scores and general well-being scores than CD patients without IBS, highlighting the inability of CDAI to differentiate between active CD symptoms and IBS-related symptoms [31]. Thus, developing PROs is a complex and meticulous process that involves patient concept elicitation interviews, expert input, and item generation to ensure their validity and clinical relevance.

This study has several strengths. It assessed adherence to objective disease monitoring and the achievement of treat-to-target goals in a real-world tertiary care setting. The cohort included both newly diagnosed and previously treated IBD patients with diverse treatment exposures, enhancing generalizability. Importantly, the study systematically captured patient-reported outcomes (PRO2 and PRO3) and evaluated their association with clinical, biochemical, and endoscopic measures of disease activity, offering insight into the utility of PROs in longitudinal disease monitoring. However, several limitations should be acknowledged. First, the retrospective design may introduce selection bias. Second, fecal calprotectin was not routinely assessed, as the test is not covered by public health insurance, limiting its inclusion as a biomarker. Third, incomplete follow-up data—particularly for endoscopic and biomarker assessments—may have led to attrition bias. To minimize this, remission rates were calculated only among patients with available data at each time point, which reflects real-world practice. Notably, a substantial proportion of patients in clinical remission opted to forgo follow-up endoscopic evaluation, especially at 1- and 2-year assessments. Due to small sample sizes at each time point, time-specific ROC analyses had limited power to determine stable cutoff values. Lastly, this study did not adjust for potential confounders such as medication exposure or disease duration, as the aim was to evaluate the unadjusted real-world relationship between PRO scores and treatment outcomes. While this approach reflects pragmatic clinical practice, it limits causal interpretation and should be addressed in future prospective studies with larger cohorts.

## 5. Conclusions

This real-world study demonstrates that patient-reported outcomes (PRO2 and PRO3) are valuable tools for monitoring IBD, particularly in clinical settings where access to biomarkers and endoscopy may be limited. Treat-to-target achievement rates were higher in UC than in CD, likely reflecting differences in disease phenotype and treatment strategies. PROs showed strong correlations with clinical remission and moderate associations with biomarker and endoscopic remission. Stratified analyses confirmed the consistent predictive performance of PROs across multiple time points. These findings suggest that PROs could serve as a simple, noninvasive tool for therapeutic disease assessment in clinical practice for both UC and CD, supporting their integration into routine care to facilitate patient-centered monitoring and timely treatment decisions.

## Figures and Tables

**Table 1:** Demographics and clinical characteristics of IBD cohort.

Characteristics	CD (*n* = 62)	UC (*n* = 50)	Total (*n* = 112)
Male gender, *n* (%)	31 (50%)	29 (58%)	60 (53.6%)
Mean age at inclusion, years (SD)	53.1 (17.3)	55.5 (15.1)	54.2 (16.3)
Mean age at diagnosis, years, (SD)	43.6 (16.5)	47 (14.4)	45.2 (15.6)
Symptom onset to diagnosis, weeks (IQR)	39.5 (13.5–81)	13 (8–26.8)	21.5 (9.8–59.5)
Disease phenotype of CD ^a^ (%)			
- A1/A2/A3	6.5%/32.3%/61.2%		
- L1/L2/L3	48.4%/14.5%/37.1%	NA	
- B1/B2/B3	74.2%/14.5%/11.3%		
Disease phenotype of UC ^b^ (%)			
- E1/E2/E3	NA	10%/22%/68%	
- S1/S2/S3		22%/38%/40%	
Upper GI involvement, *n* (%)	3 (4.8%)	NA	NA
Perianal or fistulizing disease, *n* (%)	6 (9.7%)	NA	NA
Extra-intestinal manifestation, *n* (%)	8 (12.9%)	7 (14.0%)	15 (13.4%)
Clinical score at diagnosis, median (IQR)			
HBI	5 (4–7)	NA	
Partial Mayo	NA	5.5 (4–7)	
Stool frequency (SF)	NA	2 (2–3)	
Rectal bleeding score (RB)	NA	2 (1–2)	
Biomarkers, median (IQR)			
- Hemoglobin, g/L, mean (SD)	11.3 (1.9)	11.6 (2.2)	11.4 (2.0)
- C-reactive protein (mg/dL)	33 (4–104.8)	3 (1–14.0)	6.5 (1–42.7)
- Erythrocyte sedimentation rate (mm/h)	41.5 (15.8–61.2)	32.5 (18–70)	38 (17.8–62.5)
- Albumin (g/dL)	4 (3.2–4.3)	3.9 (3.4–4.3)	4 (3.3–4.3)
Endoscopic score, median (IQR)			
- SES-CD	6 (4–12)	NA	
- MES	NA	2 (1–2)	
- UCEIS	NA	5 (4–6)	
Exposed treatment, *n* (%)			
- Mesalazine	35 (56.5%)	49 (98%)	84 (75)
- Corticosteroid	54 (87.1%)	45 (90%)	99 (88.4%)
- Budesonide	3 (4.8%)	0 (0%)	3 (2.7%)
- Salazopirine	36 (58.1%)	19 (38%)	55 (49.1%)
- Thiopurine	43 (69.4%)	35 (70%)	78 (69.6%)
- Biologics	17 (27.4%)	7 (14%)	24 (21.4%)
- Concomitant biologics and azathioprine	12 (19.4%)	7 (14%)	19 (17%)
Steroid dependent, *n* (%)	23 (37.1%)	25 (50%)	48 (42.9%)

Abbreviations: CD, Crohn’s disease; CI, Confidence interval; IBD, Inflammatory bowel disease; IQR, Interquartile range; SD, standard deviation; UC, Ulcerative colitis; SES-CD, Simple Endoscopic Score for Crohn’s disease; MES, Mayo Endoscopic Score; UCEIS, Ulcerative Endoscopic Index of Severity. Note: ^a^ Disease phenotype of Crohn’s disease was classified using Montreal classification; Age at diagnosis; A1(<17 years), A2 (17–40 years), A3 (>40 years); L1, ileal; L2, colonic; L3, ileocolonic; B1 no stricturing; B2 stricturing; B3 penetrating. ^b^ Disease location was classified using Montreal classification: E1, proctitis; E2, left-sided colitis; E3, extensive colitis. Disease severity was classified using as a partial Mayo score: S1, mild; S2 moderate; S3 severe.

**Table 2:** Achieving in treatment targets for Inflammatory Bowel Disease (IBD) treatment.

Treatment Targets	CD (*n* = 62)	UC (*n* = 50)	Total (*n* = 112)
Baseline at inclusion			
Clinical remission	47 (75.8%)	42 (84%)	89 (79.5%)
Biochemical remission	34 (54.8%)	39 (78%)	73 (65.2%)
Endoscopic remission	25 (58.1%)	25 (71.4%)	50 (64.1%)
At 1-year			
Clinical remission	42 (70.0%)	35 (79.5%)	77 (74.0%)
Biochemical remission	33 (41.7%)	33 (70.5%)	66 (53.8%)
Endoscopic remission	16 (50.0%)	20 (64.5%)	36 (57.1%)
At 2-year			
Clinical remission	29 (55.8%)	35 (81.4%)	64 (67.4%)
Biochemical remission	30 (63.8%)	36 (81.8%)	66 (72.5%)
Endoscopic remission	14 (50.0%)	15 (51.7%)	29 (50.9%)

## Data Availability

The main data are given in this article. The data are available from the corresponding author upon request.

## Supplementary Materials

The following supporting information can be downloaded at: https://www.mdpi.com/article/10.3390/jcm14134733/s1, Supplement Table S1: Calculation of Patient report outcomes (PRO), PRO2 and PRO3 in Crohn’s disease, Supplement Table S2: Trend changes in clinical score, biochemical value and endoscopic score; Supplement Table S3: Stratified ROC analyses at individual time points for PRO2 and PRO3 in Crohn’s disease; Supplement Table S4: Stratified ROC analyses at individual time points for PRO2 in UC; Supplement Figure S1: PRO2 and PRO3 for prediction of biomarker and endoscopic remission based on disease activity in Crohn’s disease patients; Supplement Figure S2: PRO2 for prediction of biomarker and endoscopic remission in UC patients with clinical remission vs. clinical activity.
