# Supplementary material for: Objective Treatment Targets and Their Correlation with Patient-Reported Outcomes in Inflammatory Bowel Disease: A Real-World Study
Source: J Clin Med. 2025 Jul 4;14(13):4733. doi: 10.3390/jcm14134733 (PMC12251468; doi:10.3390/jcm14134733)

Supplement table 1

Calculation of Patient report outcomes (PRO), PRO2 and PRO3 in Crohn's disease

PRO2

Variable	Weight factor	Total
Number of liquid or very soft stools	X 2	
Abdominal pain<br>0 = none<br>1 = mild<br>2 = moderate<br>3 = severe	X 5	
PRO2 total	=	

PRO3

Variable	Weight factor	Total
Number of liquid or very soft stools	X 2	
Abdominal pain<br>0 = none<br>1=mild<br>2=moderate<br>3=severe	X 5	
General well-being<br>0=generally well, 1=slightly under par, 2=poor, 3=very poor, 4=terrible	X 7	
PRO3 total	=	

Calculation of Patient report outcomes (PRO), PRO2 in Ulcerative colitis

Variable	Weight factor	Total
Stool frequency (SF)<br>0 = normal number of stools<br>1 = 1-2 > normal<br>2 = 3-4 > normal<br>3 = 5 > normal	X 1	
Rectal bleeding (RB)<br>0 = no blood<br>1 = streaks of blood (< half the time)<br>2 = obvious blood (most of the time)<br>3 = blood alone passed	X 1	
PRO2 total	=	

Supplement table 2. Trend changes in clinical score, biochemical value and endoscopic score

Clinical, biomark and endoscopic score	Baseline	1-year	2-year
**CD,** median (IQR)			
- HBI	0 (0,2)	0 (0,3)	1 (0,2)
- Stool frrequency score	1 (1,2)	1 (0,2)	1 (0,2)
- Abdomina pain score	0 (0,1)	0 (0,1)	0 (0,1)
- Well bening score	0 (0,0)	0 (0,1)	0 (0,0)
- PRO2	4 (2,9)	2 (0,8)	2 (0,7)
- PRO3	4 (2,11)	3 (0,9)	2 (0,7)
**Ulcerative colitis,** median (IQR)			
- pMayo	1 (0,1)	0 (0,1)	0 (0,1)
- Stool frequency	0 (0,1)	1 (0,1)	0 (0,1)
- Rectal bleeding	0 (0,1)	0 (0,0)	0 (0,0)
- PRO2	0 (0,1)	0 (0,1)	0 (0,1)
**Biomarker,** median (IQR)			
- CRP	2 (1.1 - 5.5)	2.3 (1 - 4.6)	1.8 (1 - 4.9)
- ESR	18 (8 - 46)	33.5 (12.8 - 73.5)	20 (13 - 30)
**Endoscopic score,** median (IQR)			
- SES-CD	3 (1-6)	2 (0-5)	2 (0-4)
- UCEIS	2 (1-3)	1 (1-4)	2 (1-4)

Supplement table 3:
Stratified ROC analyses at individual time points for PRO2 and PRO3 in CD

PRO2 for CD

AUC	Clinical remission	Biomarker remission	Endoscopic remission
Baseline	0.945	0.761	0.674
1-year	0.882	0.878	0.621
2-year	0.936	0.543	0.830

PRO3 for CD

AUC	Clinical remission	Biomarker remission	Endoscopic remission
Baseline	0.957	0.754	0.651
1-year	0.858	0.918	0.590
2-yaer	0.936	0.544	0.830

Supplement table 4 :
Stratified ROC analyses at individual time points for PRO2 in UC

PRO2 for UC

AUC	Clinical remission	Biomarker remission	Endoscopic remission
Baseline	1.000	0.605	0.934
1-year	0.922	0.835	0.591
2-yaer	1.000	0.748	0.847

Supplement Figure 1

PRO2 and PRO3 for prediction of biomarker and endoscopic remission based on disease activity in Crohn disease patients

PRO2 in clinical remission CD

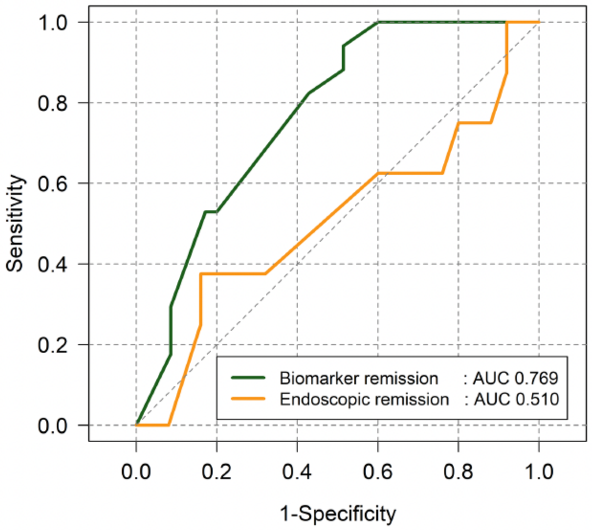

PRO2 in clinical activity CD

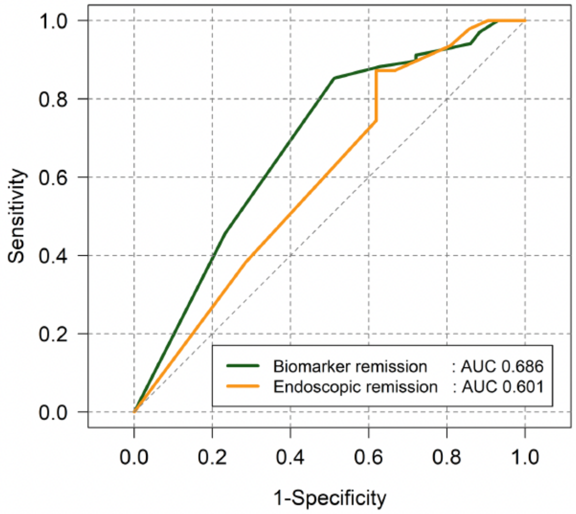

PRO3 in clinical remission CD

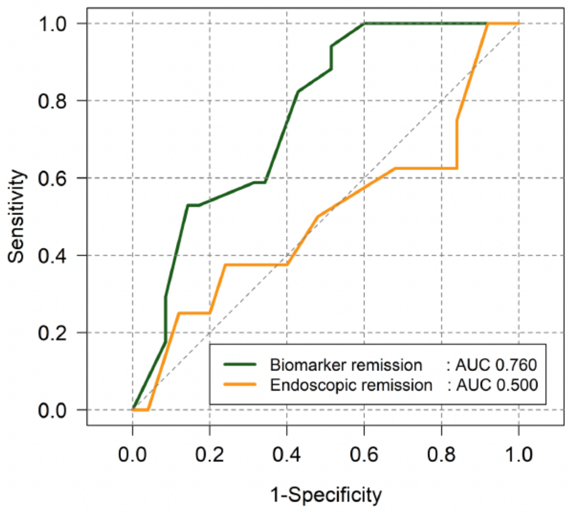

PRO3 in clinical activity CD

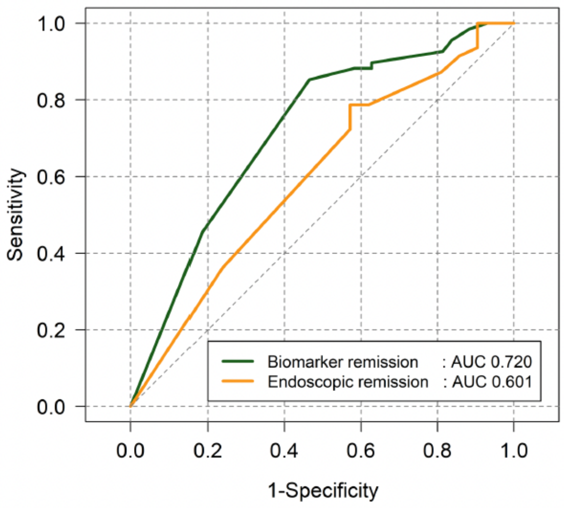

Supplement Figure 2

PRO2 for prediction of biomarker and endoscopic remission in ulcerative patients with clinic remission vs. active clinical activity

PRO2 in clinical remission

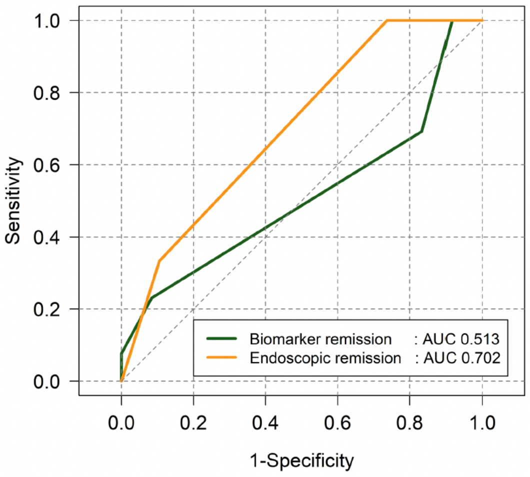

PRO2 in active clinical activty

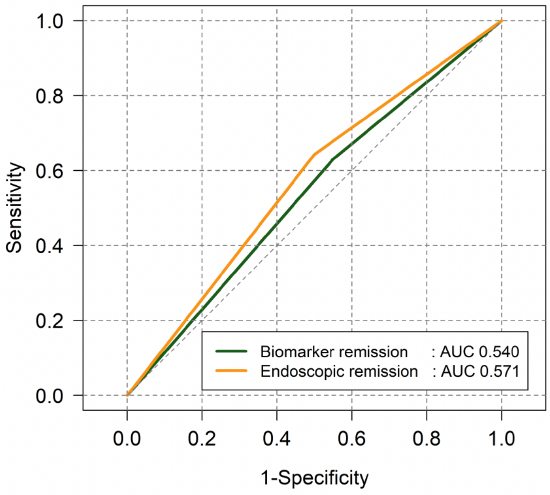

Supplement: Supplementary file 1 [file jcm-14-04733-s001.zip › jcm-3716844-supplementary.pdf]
